# Intraspecific Combinations of Flower and Leaf Volatiles Act Together in Attracting Hawkmoth Pollinators

**DOI:** 10.1371/journal.pone.0072805

**Published:** 2013-09-12

**Authors:** Zsolt Kárpáti, Markus Knaden, Andreas Reinecke, Bill S. Hansson

**Affiliations:** 1 Department of Evolutionary Neuroethology, Max Planck Institute for Chemical Ecology, Jena, Germany; 2 Plant Protection Institute, Centre for Agricultural Research, Hungarian Academy of Sciences, Budapest, Hungary; 3 Department of Behavioural Ecology and Evolutionary Genetics, Max Planck Institute for Ornithology, Seewiesen, Germany; INRA-UPMC, France

## Abstract

Insects pinpoint mates, food and oviposition sites by olfactory cues. Recognizing and localizing a suitable target by olfaction is demanding. Odor sources emit characteristic blends of compounds that have to be identified against an environmentally derived olfactory background. This background, however, does not necessarily disturb the localization of a source. Rather, the contrary. Sex pheromones become more attractive to male moths when being presented against a relevant plant background. Here we asked whether such olfactory coaction also characterizes foraging cues. The tobacco hornworm *Manduca sexta* feeds on nectar from wild tobacco *Nicotiana attenuata* and sacred datura *Datura wrightii* flowers. We tested how leaf-derived volatile blends as a background affect the moths' approach to flower blends. We found coaction when a flower blend was presented against a conspecific leaf volatile background but not when the blend was presented against volatiles emitted by the other host plant or by a non-host plant. Hence, our results reveal a species-specific coaction between flower blend and leaf volatile background. The ability to integrate information from different odor sources on one plant might provide the moth with a fine-grained analysis of food site quality.

## Introduction

Olfaction is a key modality for herbivorous insects to recognize and locate potential mates, food and oviposition sites. In moths, behavioral responses of males to sex pheromones have been well investigated [1]. The pheromone cocktail emitted by females usually contains several compounds, the ratio of which is crucial for male attraction [2], [3]. Male attraction to sex pheromones can, however, be augmented by presenting the pheromone against a relevant leaf volatile blend emitted by a suitable larval host plant [4], [5]. The male is more attracted to a female already situated on a suitable egg-laying substrate compared to one, which is not. As modified male responses to the pheromone blend at a plant background indicate, the attraction of nectar-foraging moths to flower blends may also depend on specific combinations of flower scents and vegetative plant odor background.

In order to identify potential nectar sources, a hungry insect may benefit from the ability not only to take into account the flower odor but also to consider the leaf volatile background when identifying potential nectar sources. This would further improve the resolution of the olfactory landscape.

Hawkmoths in general, and more specifically females of the tobacco hawkmoth, *Manduca sexta*, (Sphingidae), nectar feed on a wide variety of plant species from divergent plant families [6]. When foraging, they therefore encounter a series of different conditions and plant defense strategies. These may include (i) a delay between flowering and growth of vegetative plant tissues that otherwise might become subject to herbivory, (ii) defensive secondary metabolites in flower parts and nectar as well as (iii) different volatile emissions from leaves and flowers to attract pollinators and repel herbivores [7], [8]. However, nectar feeders and larval herbivores may belong to the same species as in the case of *M. sexta*. The flowers of sacred datura, *Datura wrightii*, are one of the major nectar sources for the tobacco hornworm [9], and *D. wrightii* relies on *M. sexta* as one of its main pollinators [6]. At the same time, *D. wrightii* is a highly preferred host for ovipositing *M. sexta* females [10] and tolerates herbivory to a certain extend [11]. In contrast, the much smaller wild tobacco, *Nicotiana attenuata*, plants heavily rely on direct defense by producing nicotine or anti-digestive proteinase inhibitors and indirect defense by attracting predators through feeding-induced herbivore-specific volatiles emissions [12], [13], [14]. Despite being self-compatible, *N. attenuata* may benefit from hawk moth pollinator mediated outcrossing [15]. Corresponding to the different defense strategies, *M. sexta* females prefer to oviposit on *D. wrightii* compared to *N. attenuata* [10], while flowers from both species emit highly attractive odors of different composition [15], [16]. Among numerous odorants emitted by *D. wrightii* flowers, three components were, when presented together, necessary and sufficient to attract foraging moths [16]. Only two compounds have been identified in *N. attenuata* flower headspace [15]. The system consisting of the two Solanaceae *D. wrightii* and *N. attenuata*, and the tobacco hawkmoth, *M. sexta*, thus, offers a unique opportunity to explore how vegetative plant odors may affect nectar foraging on plants that flower with fully developed leaves. More specifically we ask whether the attractiveness of flower odors is enhanced by an attractive leaf volatile background and whether a species-specific flower and vegetative odor combination is required for positive blend interaction. This investigation was carried out with young and unmated females. These are known to have a strong preference for nectar foraging as compared to a bias for egg laying related host search in mated females [17]. The moths had no previous experience with plant volatiles.

We show that the attractiveness of flower blends of *D. wrightii* and *N. attenuata* to naïve, unmated, and hungry *Manduca* females are affected by leaf odors. Although the olfactory background of a *D. wrightii* plant increased the attractiveness of the *D. wrightii* flower blend, it did not affect the attractiveness of the *N. attenuata* flower blend. Conversely, the olfactory background of a *N. attenuata* plant augmented the attractiveness of a *N. attenuata* flower blend but not that of a *D. wrightii* flower blend.

Our data thus show that flower- and leaf-derived odors act together to attract female foraging moths. This coaction is, however, restricted to intra-specific flower-plant combinations.

## Materials & Methods

### Insects

*M. sexta* larvae were reared in the laboratory on an artificial diet [18]. Female pupae were kept in an environmental chamber at 25°C with 70% relative humidity on a 16 h/8 h light/dark photoperiod. The behavioral experiments were performed with unmated females 3 days post-eclosion. Responses to plant stimuli at this age strongly depend on mating status [17]. The females were starved since eclosion, i.e., they had no previous access to any nectar source. Each individual was tested only once.

### Odor sources

**Figure 1:**
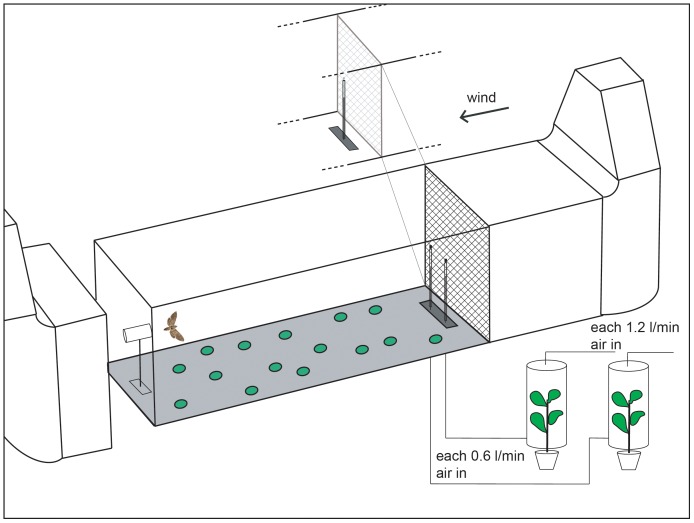
Schematic drawing of the wind tunnel (length, 250 cm; width, 90 cm; height, 90 cm). Females were released from a platform 50(distance between sources, 20 cm) were placed at the upwind entrance to the wind tunnel. These consisted of filter papers loaded with synthetic flower odors. Headspace volatiles from non-flowering plants placed in a glass cylinder outside the tunnel were released close to the source of flower volatiles.

We tested moth attraction in a no-choice assay to flower and leaf odors of the two host plants, *D. wrightii* and *N. attenuata*, and to the leaf odor of the non-host plant, *Brassica oleracea* var. Rosella (Brussels sprouts). For flower odors, we used synthetic mixtures (*D. wrightii*: benzyl alcohol 90%, ±linalool 7%, benzaldehyde 3% [16]; *N. attenuata*: benzyl-acetone 97%, benzaldehyde 3% [15], [19]). The mixtures were dissolved in mineral oil (14.2 µg/µl). 1×2 cm filter paper (Whatman, England) odor sources were prepared with 10 µl of the different synthetic mixtures. The loaded filter paper was placed at the upwind entrance of the wind tunnel (Fig. 1, for details see below). 10 µl mineral oil only were used as a solvent control. The total amount of volatiles on the filter paper corresponded to the amount used by Riffell and coworkers [16].

To test the attractiveness of the natural vegetative odors of two *M. sexta* host plant species (*D. wrightii, N. attenuata*) and one non-host species (*B. oleracea*), individual non-flowering plants were placed in a cylinder (diameter, 40 cm; height, 70 cm) outside of the wind tunnel. A stream (0.8 l/min) of purified air was pumped through the cylinder into the wind tunnel to provide the moths with the headspace of the plant. To keep the visual stimuli during the different treatments constant, the release tube for the plant headspace was hidden by a filter paper identical to that used in the experiments with flower mixtures.

In a second set of experiments, the moths had to choose between two adjacent odor sources (distance between sources, 20 cm, for plume structure as deduced from experiments with titanium tetrachloride see Movie S1) that were again placed at the upwind entrance to the wind tunnel. We first compared the attractiveness of the different host plants (both regarding flower and leaf-derived odors) and, second, tested for *D. wrightii* and *N. attenuata* the attractiveness of flower odors versus leaf-derived odors.

Finally, we compared the attractiveness of flower odors to the attractiveness of the same odor combined with leaf volatiles. We combined each flower odor with the leaf odor of its own species, with the leaf odor of the other host species, and with the leaf odor of the non-host *Brassica*.

### Wind tunnel bioassays

The behavioral activity of flower and plant odors was tested in a Plexiglas wind tunnel (length, 2.5 m; width and height, 0.9 m, Figure 1, airflow 0.4 m s-1, 0,5 Lux diffuse light, 23°C, 70% RH). Laminar airflow was created by a 1×1 m wire mesh mounted between an activated charcoal filter and the odor source. Green dots (diameter 5 cm) were placed randomly in a non-overlapping pattern on the floor to provide optomotor cues. One hour prior to behavioral testing, individual females were transferred from their rearing cages to a netted releasing tube (15×22 cm) and moved into the room that had the same light, humidity, and temperature as the wind tunnel, allowing the insects to acclimatize. For testing, the tube was placed on a release platform at the downwind entrance to the tunnel, 50 cm above the floor and 210 cm downwind from the odor source. By placing dry ice with water at the position of the odor source we confirmed that the flow is laminar and that the odor plume reaches the test animal at its starting position.

All experiments were conducted within the first 4 hours of the scotophase. Females were tested for 5 min after taking off. Plume following was characterized by casting behavior interrupted by short straight upwind flight paths within the plume (which had been visualized by smoke before the experiments started). The following behavioral responses were characterized as follows: (1) animals that followed the plume at least halfway to the source, (2) animals that contacted the source with their proboscis. When testing the animals in a choice assay, we noted which source was contacted first and counted the number of source contacts for each animal and source.

For each presented stimulus pair, we tested the significance of the first choice using the chi-squared test. The number of visits at each of both sources was analyzed using the Wilcoxon signed-rank test. We never found contradictory results from both types of analyses, i.e., when for one odor pair the analysis of the first choice revealed a preference, be it by trend or significance, the analyses of the number of visits at the source pointed in the same direction.

## Results

**Figure 2:**
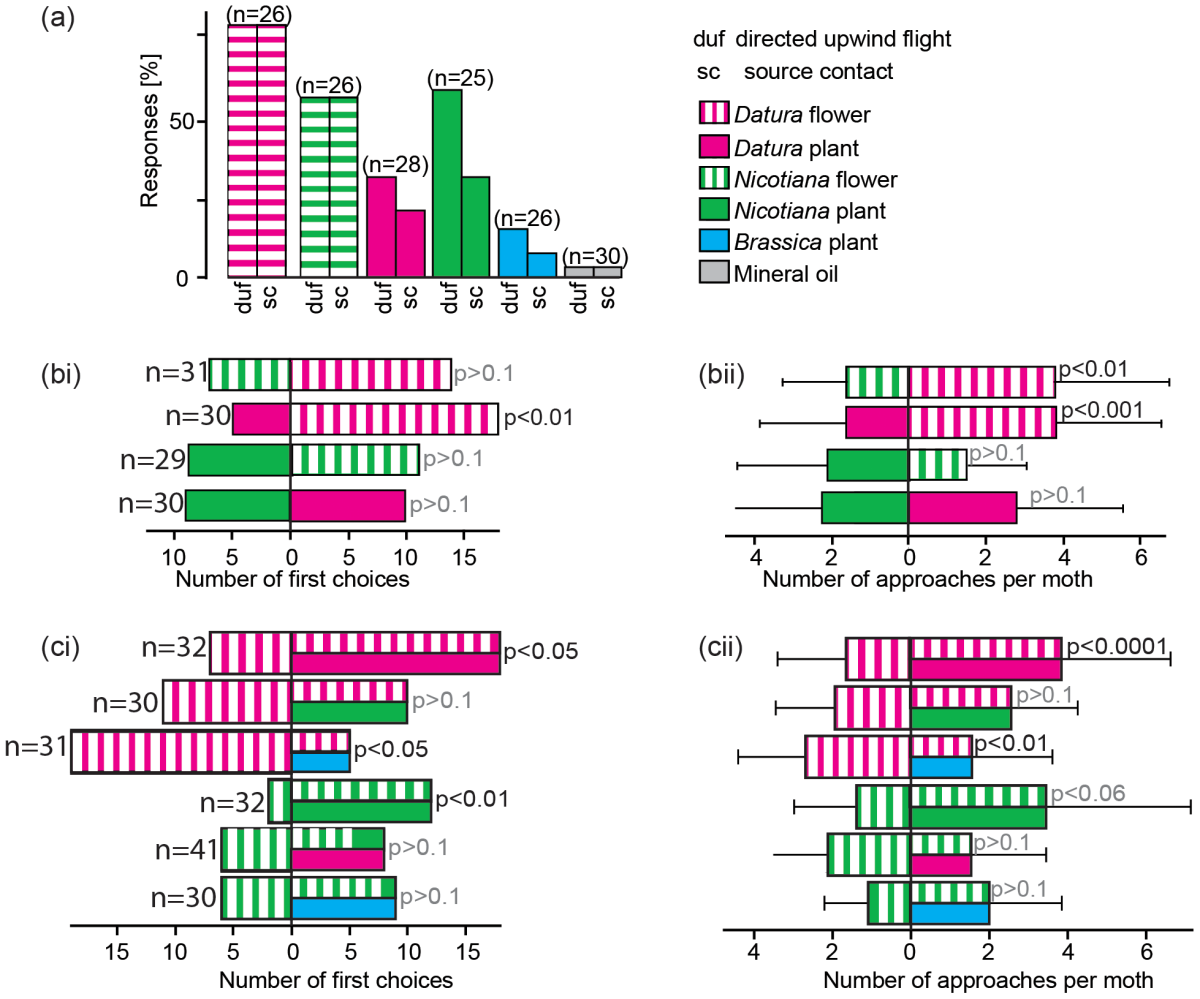
Attraction of *M. sexta* females to plant and flower odors. (a) No-choice experiment: Percentage of moths that flew upwind towards the presented odor source (duf) and reached the source with extended proboscis (sc). (b) Two-choice experiment with two single odor sources presented in the wind tunnel (20 cm apart). (b-i) Number of first source contacts. (b-ii) Total number of approaches per moth within 5 min. (c) Two-choice experiment, presenting a single flower blend stimulus and a combined flower and plant odor. (c-i) Number of first source contacts. (c-ii) Total number of approaches per moth within 5 min. Error bars depict the standard deviation.

We first tested whether moths were attracted by the uncombined flower and leaf odor blends (Fig. 2a). Most of the moths initiated upwind flights towards the flower blends and subsequently contacted the source with their proboscis, with the *D. wrightii* flower blend being more attractive than the *N. attenuata* flower blend. When offered leaf volatiles, only a few moths probed the odor source with their proboscis, irrespective of plant species. *N. attenuata* leaf odor, however, still resulted in the same number of moths flying upwind as the *N. attenuata* flower blend, while *D. wrightii* leaf odor elicited less than half as many upwind flights compared to the corresponding flower blend. Non-host *B. oleracea* leaf volatiles elicited the fewest number of flights (Fig. 2a).

The primary outcome of the no-choice experiment was confirmed when the moths had to choose between two odor sources (Fig. 2 bi–ii). The *D. wrightii* flower blend significantly outcompeted the *N. attenuata* flower blend (number of first choices (fc): p>0.1, number of approaches (na): p<0.01) and *D. wrightii* leaf odor (fc: 0.01, fa: p<0.001), while the leaf odors of *D. wrightii* and *N. attenuata* were similarly attractive (fc and fa: p>0.1). When presented adjacently, *N. attenuata* flower blend and leaf odors were equally attractive (fc and fa: p>0.1), again revealing the similarity in attractiveness of these two odor blends.

We next tested how the attractiveness of a flower blend was affected when presented against a leaf odor background (Fig. 2 ci–ii). When moths had to choose between the pure *D. wrightii* flower blend and the combination of *D. wrightii* flower blend and leaf odor, they significantly preferred the combined stimulus (fc: p<0.05, fa: p<0.0001). The same was true when the choice had to be made between pure *N. attenuata* flower blend and a combination of *N. attenuata* flower blend and leaf odors (fc: p<0.01, fa: p = 0.06). Again, the moths preferred the combined cue. However, when we presented *D. wrightii* flower blend against a background of the odor of a non-flowering *N. attenuata* plant and *N. attenuata* flower blend against a background of the odor of a non-flowering *D. wrightii* plant, we did not find any preferences for the combined cues (fc and fa: p>0.1). When flower blends were tested against a background of the non-host *B. oleracea* leaf odor, the moths' response to a combination with the *N. attenuata* flower blend equaled their response to the flower blend alone (fc and fa: p>0.1); when the odors of the *B. oleracea* plant were combined with *D. wrightii* flower odors, on the other hand, moths were significantly less attracted than they were to the flower bouquet on its own (fc: p<0.05, fa: p<0.01). In summary, attraction to a flower blend was increased only when combined with the conspecific leaf odor.

## Discussion

In most cases, odor cues important for survival and reproduction are not monomolecular but, rather, consist of mixtures of different odorants. The identity, concentration and ratio of chemical components in these mixtures are important for odor-guided behavior in numerous species of vertebrates and invertebrates. For example, only the species-specific mixture of pheromone components elicits appropriate behavioral responses in animals as divergent as mice, elephants, and moths [1], [2]. When it comes to plant volatiles, aphids have been shown to be repelled by host-plant-derived odorants when components are sensed individually, even though a mixture of the same compounds constitutes a highly attractive blend [20]. Background odors are, in turn, known to modulate the attraction to specific blends. Plant volatiles can modulate both the degree of attraction and the physiological response to sex pheromone in males of many moth species [4], [5], [6], [21]. The olfactory background against which it is sensed can thus affect the behavioral response towards an odor.

In the present study we asked how leaf and flower blends interact in nectar foraging female *M. sexta* hawk moths. Females visit plants for both nectar feeding and oviposition. Odor-based localization of flowers and potential host plants has been well described (flowers: [10], [17], [22]; plants: [13]). Plant-derived leaf odors are typically discussed in the context of host-plant localization and oviposition site choice, whereas flower odors are studied from a foraging perspective. It has been shown that a few key compounds of the *D. wrightii* flower odor blend are necessary and sufficient to provoke directed upwind flights in *Manduca* [16]. In the present study, we also show that a mixture of two components of the *N. attenuata* flower bouquet [15] provokes plume following and feeding behavior in a similar way. Flower blends are highly attractive stimuli for a hungry moth. Beyond attractiveness our data also show that the mixtures used convey odor identity to the foraging females since they were discriminated in a choice test using similar odor intensities (Fig. 2 bii).

Next we asked how leaf background odors affect the attractiveness of flower odors. In order to focus rather on foraging than on oviposition behavior, we used unmated and hungry *Manduca* females. It has been described that aged unmated *Manduca* moths sometimes show oviposition behavior and lay unfertilized eggs [23]. However, in agreement with the data reported by [17] we never observed abdomen curling, which would indicate egg-laying behavior during our experiments. On the contrary, whenever the moths contacted the odor source they extended their proboscis, indicating feeding motivation. These conditions allowed us to test whether leaf odors play a role in guiding moths in their search for nectar. As expected, fewer moths contacted the odor sources emitting leaf odors compared to flower blends in the no-choice experiments.

When we tested flower blends of *D. wrightii* and *N. attenuata* against a background of leaf volatiles emitted by conspecific plants, the moths preferred the combined odor over the flower blend alone. The behavioral activity of leaf odors is thus not restricted to oviposition, as leaf odors co-act with flower blends, thereby increasing the attractiveness of the flower blend. Raguso and Willis (2005) found in good agreement with our results that both vegetative and flower odors synergize visually guided feeding [22]. Surrogate flowers were attractive and induced proboscis extension when scented with either flower or vegetative plant odors. However, in their experiments vegetative plant odors failed to enhance the attractiveness of scented surrogate flowers. A number of factors may explain this discrepancy. Raguso and Willis (2005) performed their experiments in the open field [22]. Therefore, even the artificial flowers that were scented only with flower odor were presented against an olfactory background. Furthermore, the study was performed with wild moths that were most probably experienced. The moths' preference for flower odors is flexible and influenced by learning [24]. Wild moths may learn to associate the multimodal *D. wrightii* flower percept to a nectar reward. Furthermore, appearance, i.e. size, reflectance, and shape determine the attractiveness of visual flower stimuli [22]. While we used a small piece of filter paper as a minimalized visual stimulus these authors used true *D. wrightii* flowers or surrogate flowers of similar size. While in our experiment olfactory background was important to the moths, flower size may in a multimodal interaction framework override the importance of olfactory background information. Finally floral scent complexity might affect its attractiveness alone or when combined with vegetative plant odors. While this might be relevant when using the widely reduced *D. wrightii* floral blend, not more than the two compounds present in the *N. attenuata* blend were detected in floral headspace from the latter species [15]. Still we observed the same type of blend interaction in both species. Based on out data we therefore conclude that flower and vegetative plant odors interact in attracting nectar foraging unmated naïve *M. sexta* females. However, context and crossmodal interaction may up- or down-rank the actual behavioral relevance of the combined chemical information.

It has been shown that plumes emitted by flowers contain increased humidity [25] and CO_2_ [26] and that both cues can be used by moths to pinpoint the flower. However, this information appears to be relevant only in specific context since behavioral responses to *D. wrightii* flowers can be mimicked by widely reduced synthetic blends devoid of humidity or CO_2_. It appears that humidity and CO_2_ are complementary stimuli relevant when an insect choses among flowers of plant of, e.g. different profitability [25]. Our artificial flower blends did neither contain humidity nor CO_2_, while the headspace of the leaves probably did so. Therefore, the increased attractiveness of combined flower and leaf blends could have been caused by increased humidity or CO_2_ concentration. We, therefore, asked whether the attractiveness of a flower bouquet is augmented by any addition of leaf odors (and the corresponding humidity and CO_2_). To answer this question, we first exchanged leaf odors between *D. wrightii* and *N. attenuata* so that the “wrong” leaf odor formed the background to the flower blend. We put a “cat's head on the dog” and vice versa. In both cases, synergy was completely abolished. We conclude that not just any attractive leaf odor background will increase a flower's blend attractiveness. Only the bouquet of conspecific foliage does so. When we tested flower blends against the background of a non-host, i.e., *Brassica*, blend, the moth's response to the *N. attenuata* flower blend did not change but it decreased to the *D. wrightii* flower blend. As expected, a background of “meaningless” plant odors does not increase the willingness of a moth to respond to a flower blend and can even affect it negatively despite the presence of water vapor and CO_2_.

Foraging *Manduca* moths are exposed to a plethora of different odorants in constantly varying concentrations. The olfactory system is challenged with the task of filtering out relevant information from this universe of molecules and in so doing lay the groundwork for relevant behavioral repertoires. Monomolecular odorants emitted by nectar-providing flowers are significantly less attractive than the odorants that make up a full flower bouquet, and are thus very likely not meaningful on their own, as they could also stem from other, less optimal or even meaningless sources. The increased response to flower blends compared to individual blend constituents is therefore beneficial as it provides the moth (and the flower) with a more specific communication channel. The presence of the conspecific leaf odor further increases attraction to the flower blend. In this way, the olfactory message becomes even more relevant and the risk of mistaken attraction is further decreased. The interaction of flower and leaf blends can thus be seen as a strategy for optimizing the sensory filter and thus enhancing odor-based food source orientation.

## Supporting Information

Movie S1 **Plumes emitted by two sources (source distance, 20 cm) are separated over at least one-meter distance.** We have visualized the plume structure by the use of smoke gained from Titanium tetrachloride (TiCl4).
(MOV)
